# Supplementary material for: Molecular Typing Reveals Distinct Mycoplasma genitalium Transmission Networks among a Cohort of Men Who Have Sex with Men and a Cohort of Women in France
Source: Microorganisms. 2022 Aug 6;10(8):1587. doi: 10.3390/microorganisms10081587 (PMC9413324; doi:10.3390/microorganisms10081587)
Supplement: Supplementary file 1 [file microorganisms-10-01587-s001.zip › microorganisms-1737561-Table S2.pdf]

Table S2. Multivariate analysis for the most prevalent *mgpB* type distribution according to the sex and the macrolide resistance status.

**ST2 *vs* variables**	**Estimate (aOR)**	**95% CI**	**P-value**
Sex (male)	0.42	0.11-1.56	0.19
Macrolide resistance status (resistant)	1.53	0.66-3.58	0.32

**ST4 *vs* variables**	**Estimate (aOR)**	**95% CI**	**P-value**
Sex (male)	2.39	0.84-6.84	0.10
Macrolide resistance status (resistant)	2.23	0.91-5.45	0.08

**ST7 *vs* variables**	**Estimate (aOR)**	**95% CI**	**P-value**
Sex (male)	0.29	0.08-1.11	0.07
Macrolide resistance status (resistant)	1.01	0.42-2.43	0.98

aOR: adjusted odds ratio; 95% CI: 95% confidence interval.
