# Supplementary figures and images for: Molecular Typing Reveals Distinct Mycoplasma genitalium Transmission Networks among a Cohort of Men Who Have Sex with Men and a Cohort of Women in France
Source: Microorganisms. 2022 Aug 6;10(8):1587. doi: 10.3390/microorganisms10081587 (PMC9413324; doi:10.3390/microorganisms10081587)

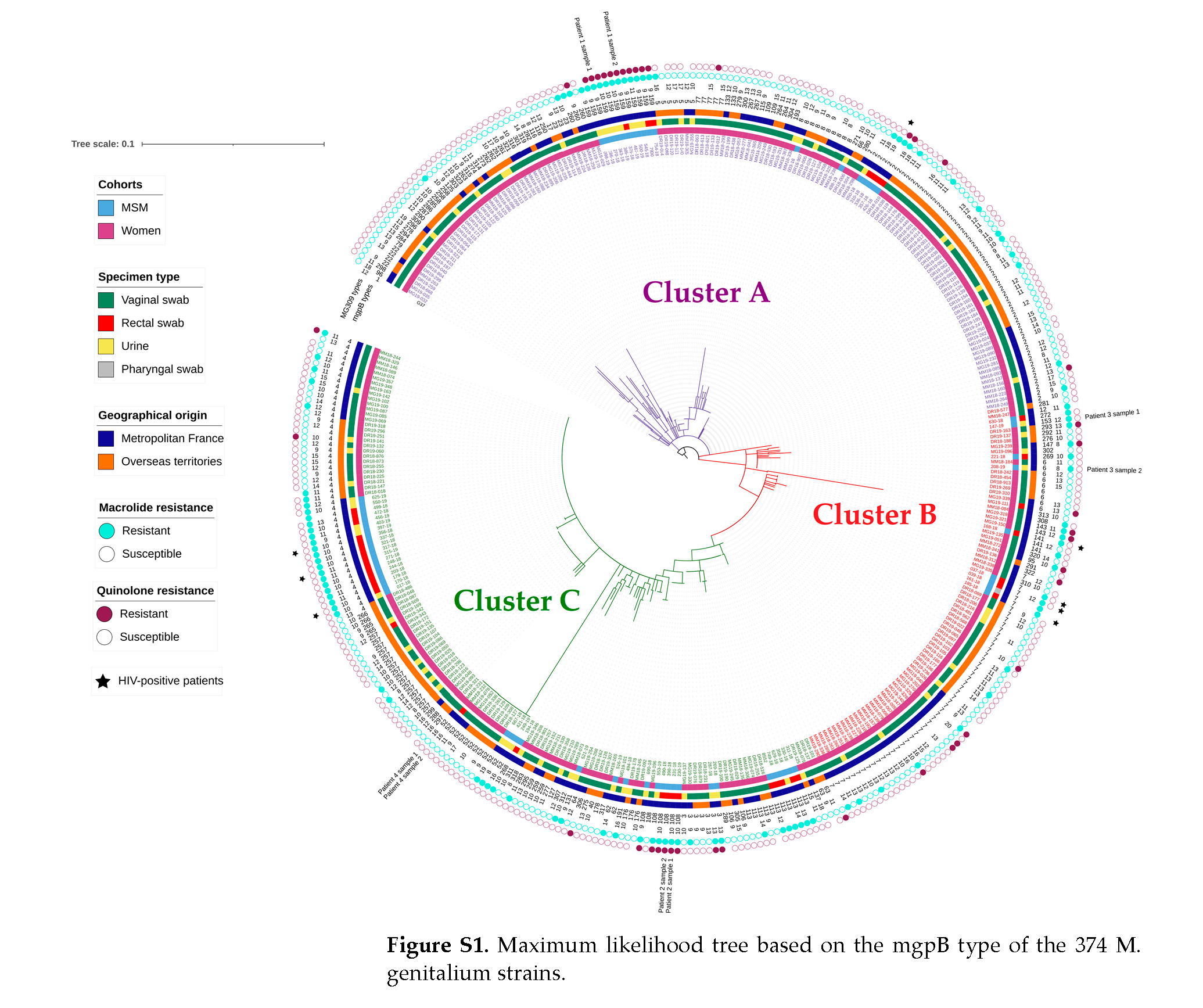

Supplement: Supplementary file 1 [file microorganisms-10-01587-s001.zip › Figure S1.png]
